# Patterns of needs among Iraqi families caring for children with autism spectrum disorder: a cross-sectional study

**DOI:** 10.3389/fpsyt.2025.1562083

**Published:** 2025-06-20

**Authors:** Nadia Kadhim Nayyef, Shatha Mohammed Jasim, Faris Lami, Osamah Abbas Jaber, Nahid Dehghan Nayeri, Mahdi Shafiee Sabet, Ghaith Al-Gburi

**Affiliations:** ^1^ Al-Subtain Academy for Autism and Neurodevelopmental Disorders, Karbala, Iraq; ^2^ Al-Subtain University of Medical Sciences, International Branch of Tehran University for Medical Sciences, Karbala, Iraq; ^3^ Department of Community Medicine, College of Medicine, University of Baghdad, Baghdad, Iraq; ^4^ Nursing and Midwifery Care Research Centre, School of Nursing and Midwifery, Tehran University of Medical Sciences, Tehran, Iran; ^5^ Department of Family Medicine, School of Medicine, Tehran University of Medical Sciences, Tehran, Iran; ^6^ School of Biosciences, Birmingham University, Birmingham, United Kingdom

**Keywords:** autism spectrum disorder, childcare, medical comorbidity, psychiatric comorbidity, educational program, family need, respite care

## Abstract

**Objective:**

Children with autism spectrum disorder (ASD) often require significant family support to carry out their daily activities. Assessing the needs of these families is important to optimize the use of the limited financial and professional resources available to them. This study aims to evaluate the needs of Iraqi families who care for children with ASD in various area and explore how these needs are related to the characteristics of child.

**Method:**

Parents of children with ASD attending Al-Subtain Academy for Autism and Neurodevelopmental Disorders were included in a cross-sectional survey between January 20, 2024, and September 9, 2024. A structured standardized questionnaire was utilized to evaluate family needs in four domains: the need for information, explaining to others, childcare needs, and professional support.

**Result:**

249 parents were interviewed, consisting of 196 (78.7%) mothers and 53 (21.3%) fathers. Over 80% of parents required support in finding information, particularly information regarding services available for their children. With in their perspective domains, support in finding reading materials about similar families and talking to them were the most requested needs, being mentioned by 40.6% and 18.1% of parents, respectively. Parents of children with comorbidities reported higher childcare and professional support needs, presumably due to a higher need for respite care and more time spent talking to teacher and therapists.

**Conclusion:**

Parents should be provided with better access to information, particularly regarding services available for their children, and more opportunities to communicate with teachers, therapists, and similar families to reduce social isolation.

## Introduction

Children with autism spectrum disorder (ASD) commonly experience challenges in their everyday lives as a result of social and communication difficulties, as well as repetitive and restrictive behaviours or interests (1). Additionally, the adaptive function of these children may be affected by cognitive problems and comorbid conditions (2, 3). Therefore, conscious effort should be made to provide these children with support for daily living. The DSM-5 acknowledges this and has categorized ASD into three levels based on the level of support needed: level 1 (requiring support), level 2 (requiring substantial support), and level 3 (requiring very substantial support) (1).

ASD is one of the most common neurodevelopmental disorders worldwide with a prevalence of around 0.72% (4). Yet, in Iraq, it largely neglected that the most commonly cited prevalence of 0.89%, reported in a previous field review from the northern region, is based on prediction from the World Population Review, rather than epidemiological surveillance (5). The same field review cited the lack of formal diagnostic standards and the lack of electronic health records as persisting challenges against evidence-based healthcare provision and medical research in the region.

In Iraq, ASD is diagnosed by a pediatrician or a psychiatrist based on the criteria described in the 11^th^ edition of the International Classification of Diseases (ICD-11) or the 5^th^ edition of the Diagnostic and Statistical Manual of Mental Disorders (DSM-5) with a wide range of variability across centers. Similar to surrounding countries, stigmatizing cultural beliefs, including the notion that children’s behavioral issues are caused by violating religious taboos, act as barriers that defer parents from actively seeking a diagnosis of their children (6). The lack of locally validated assessment tools has also been discussed as an important barrier for early diagnosis and intervention (5).

Once diagnosed, Iraqi children tend to receive healthcare services that mostly focus on providing basic daily needs. Speech and occupation therapy can also be provided by both public and private institutions (5). However, these centers are scarce compared to high-income countries and are not available in rural areas. Children with high-functioning ASD can also be enrolled in mainstream schools (both public and private), while those with more severe symptoms might require special education services which have limited availability (7).

Previous studies on the impact of having a child with ASD on the family have been conducted both in western and surrounding middle eastern countries. These studies have shown that the vast majority of support for these children tend to be provided by family members (8), leading to stress (9, 10), financial burdens due to medical and non-medical expenses (11, 12), and social burdens in the form of self and enacted stigma (13). As noted in a previous qualitative study from Iran, these families have complex needs that span a wide range of domains, including obtaining information, explaining their child’s condition to others, financial aid, access to mental health and childcare services, etc (14). These needs are similar, in some respects, to the patterns of needs described by parents of children with other developmental disabilities and chronic health conditions in general (15–17).

Given the limited financial and professional resources available to Iraqi children with ASD and their families, it is vital to evaluate patterns of family needs as it allows us to improve the utilization of these resources. In Iraq, previous studies have shown the psychological impact of having a child with ASD (10, 18). However, to our knowledge, none has been conducted to assess the patterns of family needs. Addressing the needs of these families not only helps improve the children’s adaptive outcomes and the parents’ quality of life but can also conserve healthcare resources in the long run by reducing visits that arise from failure to address these issues early on.

This study aims to explore the needs of Iraqi families caring for children with ASD across various domains, such as the need for information, explaining to others, childcare needs, and professional support. The relationship between family needs and the child’s characteristics was also investigated.

## Materials and methods

### Design

As part of a research collaboration between Al-Subtain University of Medical Sciences and Al-Subtain Academy for Autism and Neurodevelopmental Disorders, a cross-sectional study was conducted between January 20, 2024, and September 9, 2024.

Data was collected from parents attending with their children at Al-Subtain Academy from April 10, 2024, to June 10, 2024. This healthcare center was selected for the study as it is the only center within the province of Karbala that provides specialized speech, behavioural, and occupational therapy for children with ASD from the province and the surrounding middle and southern regions of Iraq.

### Eligibility criteria

In order to be considered for inclusion in the study, children had to receive a diagnosis of ASD through a clinical evaluation by a consultant in children and adolescent psychiatry having at least 10 years of experience in general psychiatry and at least 5 years in working with children with ASD. The DSM-5 criteria were used to guide the evaluation process, which consisted of an interview with the child’s parent and direct observation of the child’s behaviour. The interview was adjusted according to the chief complaint. However, all parents were asked about their child’s adaptive function, developmental milestones, vaccination history, and birth events. Children were observed to assess their activity level, mood, motor behaviours, social interaction, communication, and type of play. The children were also evaluated for the presence of comorbid conditions and this information was recorded in the diagnostic report at the end of the diagnostic appointment.

For children with ASD, parents tend to be responsible for addressing the majority of the child’s needs, including assistance with feeding, dressing, and using the toilet. Therefore, only parents were included in the study, providing a more precise understanding of the impact of ASD on family needs. Other family members who accompanied the child but were not primary caregivers were not included, as they may be less familiar with the overall family needs compared to the parents.

### Participants

A total of 249 parents were interviewed, with 196 mothers (78.7%) and 53 fathers (21.3%). **Table 1** illustrates that the children had a median age of 5 years, with an IQR from 3.75 to 6 years old. Of these children, 189 (75.9%) were male, and 114 (45.8%) had other comorbid conditions in addition to ASD. The most common additional conditions were ADHD (82, 32.9%), intellectual disability (19, 7.6%), and epilepsy (6, 2.4%).

**Table 1:** Sample characteristics (N = 249).

Characteristic	Distribution
Parent demographics	
Age (Years) (Median, IQR)	34 (30 – 41)
Relation to the child: Mother (N, %)	196 (78.7)
Marital status (N, %)	
Married	238 (95.6)
Divorced/widowed	11 (4.4)
Educational level (N, %)	
No formal education	16 (6.4)
Primary school	37 (14.9)
Secondary school	75 (30.1)
Bachelor or above	121 (48.6)
Family income (N, %)	
Poor	12 (4.8)
Average	164 (65.9)
Good	73 (29.3)
Residence (N, %)	
Urban	212 (85.1)
Rural	37 (14.9)
Consanguinity: Yes (N, %)	93 (37.3)
Number of children (Median, IQR)	3 (2 – 4)
Number of children with ASD (N, %)	
One	229 (92.0)
Two	20 (8.0)
Child demographics	
Age (Years) (Median, IQR)	5 (3.75 – 6)
Gender: Male (N, %)	189 (75.9)
Comorbid conditions: Yes (N, %)	114 (45.8)

### Measures

Data was collected using a structured questionnaire divided into two sections (see research questionnaire). The first section included eight items to inquire about the parent’s demographic characteristics, such as the age (in years), relation to the child (mother or father), marital status, education level, residence, number of children in the family and number of children with ASD, and whether the parents were related through a consanguineous marriage. Additionally, four items related to the child’s demographics were included (age, gender, presence and type of comorbid conditions as per the psychiatrist diagnostic report).

The second section included items from the revised version family needs survey (FNS-R). The original survey was developed in 1988 to assess the needs of families with handicapped infants using 35 items (19, 20). Previous studies have shown moderate to good test-retest reliability (ICC = 0.67-0.81), internal consistency (α = 0.65-0.86) for the different domains of needs (21, 22). The revised version has been used to investigate the needs of families, where each item is presented as a 3-item Likert scale where a rating of 1 indicates that support is not needed on this item, 2 indicates uncertainty, and 3 indicates that support is needed (23). There are thirty-five items arranged across seven domains. Based on feedback from a pilot study of 25 parents, two domains were excluded: financial needs and family functioning/interpersonal social needs. Parents expressed that these items should not be inquired about as they are not expected to be provided by the local healthcare system. Additionally, one item from the professional support domain concerning “the need to meet a minister or a priest” was omitted as it was deemed inappropriate to meet with a government official in our local setting. As such, five domains were included in our study: need for information (7 items), explaining to others (5 items), childcare needs (3 items), community services (3 items), and professional support (2 items), all with good internal consistency as indicated by Cronbach’s α values of >0.7 (24) (**Table 2** **).**

**Table 2:** Internal consistency of the study scales (N = 249).

Family needs scales	Alpha Cronbach
Need for information	0.991
Explaining to others	0.958
Childcare needs	0.733
Community services	0.860
Professional support	0.804

### Procedure

Data was collected during a 20 to 30-minute interview between the child’s parent and a consultant pediatrician with more than 3 years of experience in working with parents of children with ASD. During the interview, the parents’ and the child’s demographic information were collected first followed by an inquiry on the required family needs.

### Data quality assurance

Missing values were prevented by instructing the pediatrician to complete all the fields during the data collection interview. Reducing the impact of confirmatory bias was achieved by not analyzing the data until after data collection had ended and by ensuring that data was collected by a consultant pediatrician who was not involved in the child’s diagnostic evaluation and no prior contact with the child and their family. In order to minimize response bias, parents were reassured that they have the freedom to participate, withdraw, or refuse from the study at any time. They were also informed that their honest responses would not affect the services their child received, as all data collected was anonymous. This was done to reduce the potential of self-desirability bias. Finally, the STROBE guideline, which has received approval from the Equator Network for reporting results of cross-sectional surveys, was employed to minimize reporting bias (25).

### Statistical analysis

The Statistical Package for Social Sciences (SPSS) program version 28 was utilized for data analysis. Normality was examined through the Shapiro-Wilk and D’Agostino-Pearson K^2^ tests, which revealed a non-normal distribution for all continuous variables in the study (**Table 3**) (26). As a result, the median and interquartile range (IQR) were used to describe continuous variables, while categorical variables were summarized using proportions. Additionally, GraphPad Prism version 10 was employed to visualize the data in this study.

**Table 3:** Normality testing for the study variables.

Variables	Shapiro-Wilk			D’Agostino-Pearson K^2^	
	Statistic	df	P-value	Statistic	P-value
Parent’s age (years)	0.980	249	**0.001**	7.109	**0.029**
Number of children	0.898	249	**<0.001**	22.719	**<0.001**
Child age (Years)	0.961	249	**<0.001**	7.116	**0.028**
Need for information	0.976	249	**<0.001**	94.562	**<0.001**
Explaining to others	0.472	249	**<0.001**	53.485	**<0.001**
Childcare needs	0.694	249	**<0.001**	71.090	**<0.001**
Community services	0.694	249	**<0.001**	126.661	**<0.001**
Professional support	0.474	249	**<0.001**	47.340	**<0.001**

Statistically significant results are highlighted with bold text.

The intercorrelation between the family needs scales as well as the correlation between the child’s age and family needs scales were tested using Spearman’s rank correlation. The associations between family needs and the child’s gender and comorbidity were tested using the Man-Whitney U test. Within each family needs scale, the Benjamin Hochberg procedure for multiple testing was used to reduce the false discovery rate (FDR) to 5% (27).

The chi-square test was utilized to examine the relationship between individual items (from professional support and childcare domains) and the presence of comorbidity. To facilitate this, the categories “Support is not needed” and “Unsure” were combined into one group and compared against the category “Support is needed”. Yate’s continuity correction was also conducted as a more conservative approach to association testing compared to Pearson’s chi-square. For cases where the expected frequency was <5, the Fisher-exact test was utilized and supplemented with Boschloo’s test. The latter utilizes Fisher’s exact p-value as a test statistic and provides a modification with higher statistical power (28). For all statistical tests mentioned, a p-value of 0.05 was considered significant.

## Results

### Needs among families of children with ASD

**Figure 1:**
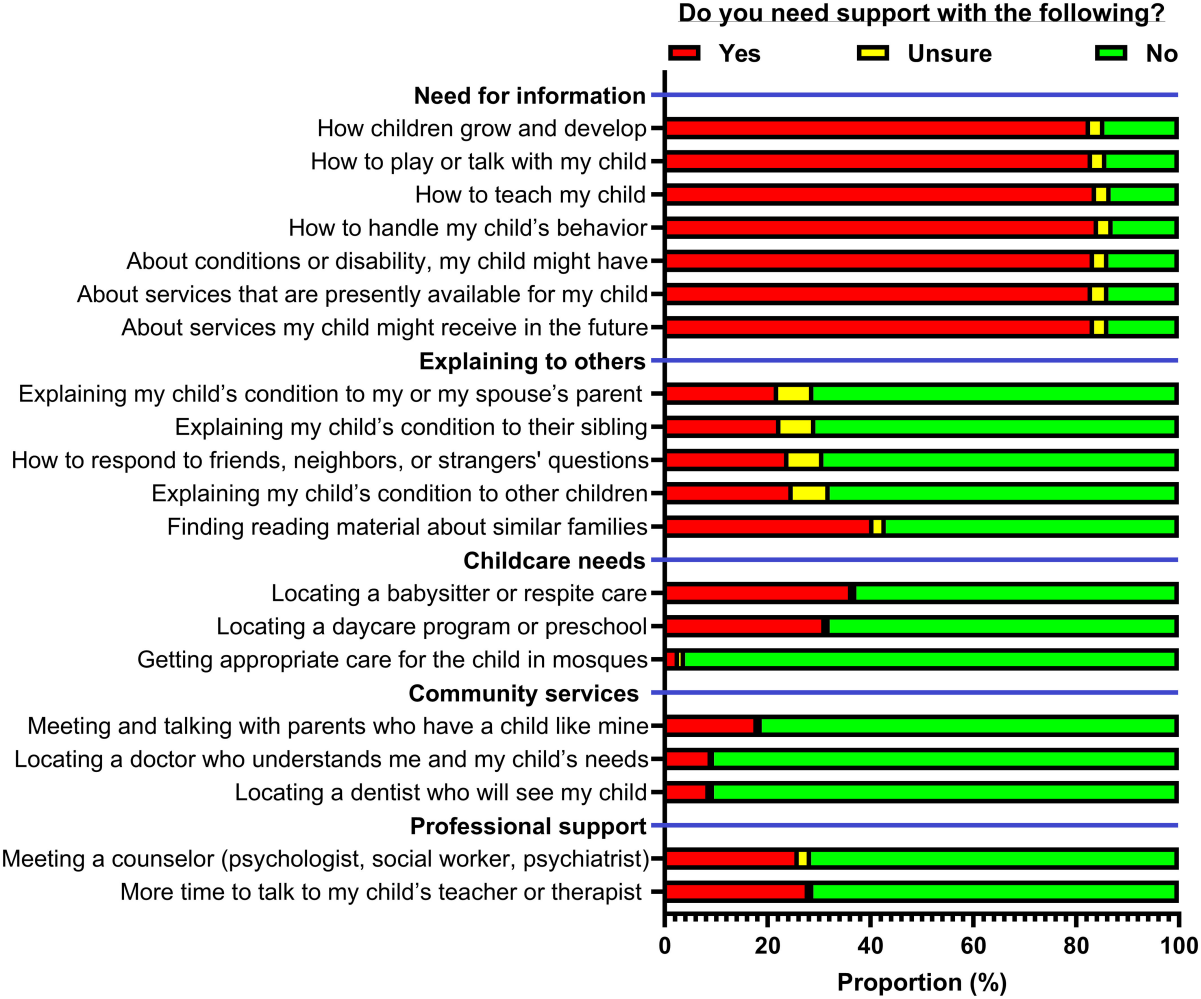
Family needs assessment among parents of children with autism spectrum disorder (N = 249).

Parents’ responses to the family needs survey are summarized in **Figure 1**. More than 80% of parents indicated that support is needed on items related to the need for information. Specifically, 210 parents (84.3%) indicated that they require more information on how to handle their child’s behaviors, 209 (83.9%) required information on how to teach their child, and 208 (83.5%) wanted to know more about the services that might be received in the future. 101 parents (40.6%) indicated that they required support in finding reading materials with similar families, and 45 (18.1%) indicated that they would like more time to meet and talk to parents with similar children. Finding a babysitter or respite care was the most requested childcare need, with 91 parents (36.5%) requiring this need, while finding appropriate care for the child at a mosque was the least required, with only 8 parents (3.2%) needing assistance with that. **Table 4** shows that the scores of the family needs scales had significant correlations with each other, particularly childcare and professional support needs, which had a strong statistically significant correlation of 0.672 (p-value = 4.6*10^-34^).

**Table 4:** Correlation between family needs scales (N = 249).

Domain	NI	EO	CN	CS	PS
NI					
EO	0.277***				
CN	0.165**	0.471***			
CS	0.085	0.363***	0.494***		
PS	0.150*	0.592***	0.672***	0.385***	

NI, Need for information; EO, Explaining to others.

CN, Childcare needs; CS, Community services.

PS, Professional support.

* P-value <0.05, ** P-value <0.01, ***P-value <0.001.

### Association between family needs and children’s characteristics

Statistically significant associations were demonstrated between the presence of comorbid conditions and family need scores (**Table 5**), specifically childcare needs (adjusted P-value = 0.024) and professional support needs (adjusted P-value = 0.030). Within these domains of family needs, statistically significant differences in the proportion of parents indicating need for support in finding a babysitter or respite care (45.6% vs. 28.9%) in the proportion of parents needing more time to talk to their child’s therapist or teacher (35.1% vs. 22.2%) (**Table 6** **).** Meanwhile, the difference in the proportion of parents indicating needing support with meeting a counselor was significant only prior to continuity correction (Adjusted p-value = 0.051).

**Table 5:** Association between family needs and child’s characteristics (N = 249).

Family needs	Age		P-value^a^	Adjusted P-value^c^
	Correlation coefficient			
Need for information	0.014		0.826	0.826
Explaining to others	-0.016		0.797	0.797
Childcare needs	-0.005		0.941	0.941
Community services	-0.055		0.384	0.384
Professional support	0.006		0.919	0.919
Family needs	Gender		P-value^b^	Adjusted P-value^c^
	Male Mean Rank	Female Mean Rank		
Need for information	123.16	130.78	0.299	0.704
Explaining to others	126.08	121.61	0.645	0.797
Childcare needs	127.98	117.52	0.288	0.432
Community services	126.98	118.75	0.266	0.384
Professional support	125.58	123.18	0.791	0.919
Family needs	Comorbidity		P-value^b^	Adjusted P-value^c^
	Yes Mean Rank	No Mean Rank		
Need for information	122.53	127.09	0.469	0.704
Explaining to others	127.38	122.99	0.598	0.797
Childcare needs	136.48	115.30	**0.008**	**0.024**
Community services	128.34	122.18	0.332	0.384
Professional support	135.91	115.79	**0.010**	**0.030**

a Spearman’s Rank Correlation was used for analysis with 0.05 as the cut-off point for significance.

b Man-Whitney U test was used for analysis with 0.05 as the cut-off point for significance.

c Within each family needs scale, the Benjamin Hochberg procedure was used to adjust the P-value for multiple testing.

Statistically significant results are highlighted with bold text.

**Table 6:** Association between childcare needs and professional support items and comorbidity of children with ASD (N = 249).

Family needs	Comorbidity			
	Yes 114 (%)^a^	No 135 (%)^a^	P-value^b^	Robust P-value^d^
Childcare needs				
Locating a babysitter or respite care	52 (45.6)	39 (28.9)	**0.006**	**0.009**
Locating a daycare program or preschool	41 (36.0)	37 (27.4)	0.147	0.189
Getting appropriate care for the child in mosques	4 (3.5)	3 (2.2)	0.706^c^	0.703^e^
Professional support				
Meeting a counselor (psychologist, social worker, psychiatrist)	37 (32.5)	28 (20.7)	**0.036**	0.051
More time to talk to my child’s teacher or therapist	40 (35.1)	30 (22.2)	**0.024**	**0.035**

a Counts and column percent are described as individuals who have answered “Yes” on the indicated items.

b Chi-square test was used for analysis with a 0.05 cut-off for statistical significance.

c Fisher’s exact test was used for analysis with a 0.05 cut-off for statistical significance.

d Yate’s continuity correction was used for analysis with a 0.05 cut-off for statistical significance.

e Boschloo’s test was used for analysis with a 0.05 cut-off for statistical significance.

Statistically significant results are highlighted with bold text.

## Discussion

More than four-fifths of the parents indicated that support is needed for each of the seven items related to the need for information, making it the most requested domain of family need in our study. This is consistent with patterns of family needs covered in previous international studies on parents of children with ASD and children with other chronic conditions in general (15–17, 23, 29). One of the studies focused on the needs of Canadian families with school-aged children with ASD and showed that most parents indicated that it was crucial for other children to understand their child’s behaviour (29). These results differ from our own findings, as only around a quarter of parents in our study indicated a need for support in explaining their child’s condition to other children (**Figure 1**). Few studies have shown a connection between family needs and the child’s age. For example, a study from China found a difference in family needs between parents of children below and above 6 years of age, coinciding with the start of elementary school (15, 23, 30). In our study, the limited age range of children, with only 4.4% being older than 6 years, may be the reason why these patterns and associations were not evident. Future studies should include families with children across various age groups to more thoroughly examine the presence of this association.

Children with ASD tend to struggle with accessing healthcare services even more than children with other developmental and mental health conditions (31). A previous systematic review from the UK has shown that a lack of knowledge about available services is a major barrier to accessing care for these families (32). Additionally, a study has shown that the time needed to navigate these services can prevent American parents from engaging in other productive activities (33). In our study, 83.1% of parents required support in obtaining information on currently needed services, and 83.6% indicated a need for information on future services. This is consistent with results from previous qualitative and quantitative international studies conducted among parents of children with ASD and other chronic conditions (14–17, 23, 29, 30). Parents training and education campaigns might increase service utilization. However, these campaigns might be less useful for Iraqi parents from rural areas and lower socioeconomic backgrounds as a previous field report discussed that specialized autism care centers might only be available in affluent areas (5). As such, rather than a top-down approach for information delivery, Iraqi parents might benefit more from information delivery via discussions that also include navigating individualized issues related to service accessibility.

Parents of children with ASD tend to experience higher rates of isolation as a consequence of enacted stigma, worsened by the prevalent misconception that ASD is caused by negligent parents, leading to self-blame and withdrawal from social situations out of fear of being unable to handle their child’s behaviour (34, 35). Previous studies from China and Sweden have shown that Group educational programs and providing parents with reading materials, highlighting the daily lives and emotional moments experienced by similar families, have reduced parents’ psychological stress and sense of isolation (36, 37). For Iraqi parents, the most requested needs, within their perspective domains, were support in finding reading materials about and talking to parents with similar children. Future interventional studies should be conducted to assess the adaptability of these practices in our local setting. Given that the different local circumstances, daily experiences of Iraqi parents might be vastly different from their western counterparts. As such, preparing reading materials based on a more systematic understanding of the experience of the Iraqi parents might be more successful than adopting materials from countries with different cultural backgrounds. More recently, studies have also examined the potential of including these elements as a part of serious games. The role of alternative reality storytelling games, in particular, has shown promising results (38).

Locating respite care was the most requested childcare need. In the context of ASD, respite care refers to the short-term care provided by an institution, or more commonly by other family members, to allow the primary caregiver a period of relief (39, 40). A study conducted in Canada found that 57% of parents considered respite care essential and identified it as their most significant unmet need (23). In our study, it is uncertain what proportion of the need for respite care was unmet. However, the fact that over a third of the parents surveyed expressed a need for assistance in finding respite care highlights the importance of addressing this issue. The lower recognition of this need among parents in our study, compared to Canadian parents, may be due to the emphasis on family role over institutional support in our local cultural background. This emphasis might act as a potential barrier to the utilization of respite care services and previous research, from Sweden, indicates that some parents experience guilt when utilizing respite care services, which can increase their stress levels (41). A more effective implementation of these services should, therefore, take into account local cultural values and present respite care as a service that supports family care rather than being a replacement. A more collaborative model for respite care, involving both family members and institutional caregivers, should also be considered.

A previous systematic report has established that stress related to child behaviours, particularly aggression, was among the most common motivations behind seeking respite care (40). Comorbid conditions have been shown to increase parents’ burden by exacerbating the behaviours associated with ASD (42, 43). As such, it is not surprising that families with children who have comorbid conditions are more likely to seek respite care. However, the presence of challenging behaviours itself might act as a barrier to accessing healthcare (32). As such, it is essential that respite care personnel receive continuous training in the management of both ASD and common comorbid conditions to allow effective implementation of these services in a local setting. A 2020 study from Israel reported that the presence of comorbid conditions in these children further complicates the diagnosis and the management of ASD (44). Therefore, it is important to provide these parents with more time to discuss matters related to their children with teachers and therapists.

It should be noted that, concerning comorbid conditions, only 7.6% of the children in our sample were classified as having intellectual disability. This is lower than the 16.7% to 84% range described in previous studies and might be due to the previously discussed lack of formal standards in assessing Iraqi children with ASD combined with scarcity of comprehensive IQ testing capacity for these children in lower- and middle-income countries including Iraq (5, 45). IQ measurement in children with ASD is particularly challenging, with discrepancies shown between older and newer studies and between different assessment tools (46, 47). In these circumstances, the limited IQ testing capacity might be prioritized for children with more severe behavioral profiles, who might be more likely to benefit from intellectual assessment.

### Strengths, limitations, and recommendations

This study was the first to address the needs of families caring for children with ASD in Iraq. Issues related to non-normal distribution of the continuous variables were addressed by conducting our analysis using nonparametric statistics. Additionally, the Benjamin-Hochberg procedure was used to reduce the false discovery rate down to 5%, thereby addressing issues related to multiple testing.

Regardless, limitations can be detected and should be addressed in future studies. First, data was collected from parents who attended the healthcare center and might not reflect the needs of parents who are less likely to seek healthcare services. Specifically, fathers might be less likely to attend healthcare centers and future studies should include both parents for the same child to give a more comprehensive view of the family needs. Second, although steps were taken to reassure parents that their responses were completely anonymous and would not affect their child’s care, self-reporting measures might still be sensitive to social desirability bias. Third, financial and social interpersonal needs domains were omitted as parents indicated that the items were inappropriate. Due to the sensitivity of discussing financial needs, especially among individuals from Arabic cultures, the revised family needs scale format of directly inquiring whether support is required to pay for certain services might not be locally appropriate. Future studies should utilize indirect scales or non-self-reported measures to assess these needs. Fourth, due to convenient sampling, generalization cannot be established with absolute certainty. Fifth, only 4.4% of the children were above the age of 6 years. As such, the needs of families with older children could not be investigated. Finally, this study did not differentiate between met and unmet family needs.

## Conclusion

In our study, most Iraqi parents required support in finding information, especially on current or future services for their children. To improve the utilization of healthcare services, training and educational campaigns should be organized to inform parents of available services while also navigating issues related to accessibility to these services. Providing parents with reading materials of similar families might help combat social isolation. However, these efforts should be based on a more thorough understanding of the daily experience of Iraqi parents rather than adopting material from different cultural backgrounds. Similarly, although a third of parents required support in finding respite care, these services should be implemented taking into account local cultural values, especially the emphasis on the role of family in providing childcare. A more collaborative approach, involving both family members and institutional caregivers, should also be considered. The presence of common comorbid conditions should also be taken into consideration while planning and implementing childcare and professional support services.

## Data Availability

The dataset used in this study can be assessed through Mendeley’s data and will be made publicly available at: https://doi.org/10.17632/9jy9937xmf.1.
