# Supplementary material for: Patterns of needs among Iraqi families caring for children with autism spectrum disorder: a cross-sectional study
Source: Front Psychiatry. 2025 Jun 20;16:1562083. doi: 10.3389/fpsyt.2025.1562083 (PMC12226537; doi:10.3389/fpsyt.2025.1562083)
Supplement: Supplementary file 1 [file Table1.docx]

**Research questionnaire (English version)**

**First:** Demographics

| **Age (years)** |  |
| --- | --- |
| **Relation to the child** | 🞎 Father 🞎 Mother |
| **Marital status** | 🞎 Married 🞎 Divorced/widowed |
| **Education** | 🞎 No formal education  🞎 Elementary school  🞎 Middle school  🞎 High school  🞎 Bachelor or above |
| **Residence** | 🞎 Urban 🞎 Rural |
| **No. of children** |  |
| **No. of autistic children** |  |
| **Consanguinity** | 🞎 Yes 🞎 No |
| **Child age (years)** |  |
| **Child gender** | 🞎 Male 🞎 Female |
| **Presence of comorbid conditions** | 🞎 Yes 🞎 No |
| **Type of comorbidity** |  |

**Second:** Family needs scale (FNS)

| **Do you need support in getting information on each of the following?** | **No** | **Not sure** | **Yes** |
| --- | --- | --- | --- |
| How children **grow and develop** | 🞎 | 🞎 | 🞎 |
| How to **play or talk** with my child | 🞎 | 🞎 | 🞎 |
| How to **teach** my child | 🞎 | 🞎 | 🞎 |
| How to handle my child’s **behaviour** | 🞎 | 🞎 | 🞎 |
| Information about **any condition or disability** my child might have | 🞎 | 🞎 | 🞎 |
| Information about **services** that are presently available for my child | 🞎 | 🞎 | 🞎 |
| Information about services my child might receive **in the future** | 🞎 | 🞎 | 🞎 |
| **Do you need support with each of the following** | **No** | **Not sure** | **Yes** |
| Explaining my child’s condition to **my parents or my spouse’s parents** | 🞎 | 🞎 | 🞎 |
| Explaining my child’s condition to **his or her siblings** | 🞎 | 🞎 | 🞎 |
| Knowing how to respond when **friends, neighbors, or strangers** ask questions about my child | 🞎 | 🞎 | 🞎 |
| Explaining my child’s condition to **other children** | 🞎 | 🞎 | 🞎 |
| Finding **reading material** about other families who have a child like mine | 🞎 | 🞎 | 🞎 |
| Locating **babysitters or respite care** who are willing and able to care for my child | 🞎 | 🞎 | 🞎 |
| Locating **a daycare program or preschool** for my child | 🞎 | 🞎 | 🞎 |
| Getting appropriate care for my child in **a mosque** | 🞎 | 🞎 | 🞎 |
| **Meeting & talking** with other parents who have a child like mine | 🞎 | 🞎 | 🞎 |
| **Locating a doctor** who can understand me and my child’s needs | 🞎 | 🞎 | 🞎 |
| **Locating a dentist** who will see my child | 🞎 | 🞎 | 🞎 |
| Meeting with a **counselor (psychologist, social worker or psychiatrist)** | 🞎 | 🞎 | 🞎 |
| More time to talk to my child’s **teacher or therapist** | 🞎 | 🞎 | 🞎 |

**Research questionnaire (Arabic version)**

**الجزء الاول:** معلومات الاهل و الطفل

|  | **العمر الاب او الام (سنوات)** |
| --- | --- |
| الاب 🞎 الام 🞎 | **العلاقة مع الطفل** |
| مطلق او ارمل🞎 متزوج 🞎 | **الحالة الاجتماعية** |
| لا يوجد تعليم رسمي🞎  مدرسة ابتدائية🞎  مدرسة الثانوية🞎  بكالوريوس أو أعلى🞎 | **التعليم** |
| ريف 🞎 مدينه 🞎 | **السكن** |
| سيئة🞎 متوسطة 🞎 جيدة 🞎 | **الحاله الاقتصادية للعائلة** |
|  | **عدد الاطفال** |
|  | **عدد الاطفال المصابين بالتوحد** |
| لا🞎 نعم 🞎 | **زواج اقارب** |
|  | **عمر الطفل (سنوات)** |
| بنت 🞎 ولد 🞎 | **جنس الطفل** |
| لا 🞎 نعم 🞎 | **وجود امراض مصاحبه** |
|  | **نوع المرض** |

**الجزء الثاني:** احتياجات العائلة

| لا | غير متاكد | نعم | **هل تحتاج الى مساعدة في الحصول على المعلومات في ما يخص كل مما يلي:** |
| --- | --- | --- | --- |
| 🞎 | 🞎 | 🞎 | كيف سينمو طفلي ويتطور |
| 🞎 | 🞎 | 🞎 | كيف ألعب أو أتحدث مع طفلي |
| 🞎 | 🞎 | 🞎 | كيف أعلم طفلي |
| 🞎 | 🞎 | 🞎 | كيف أتعامل مع سلوك طفلي |
| 🞎 | 🞎 | 🞎 | معلومات حول أي حالة أو إعاقة قد يعاني منها طفلي |
| 🞎 | 🞎 | 🞎 | معلومات حول الخدمات المتوفرة حاليًا لطفلي |
| 🞎 | 🞎 | 🞎 | معلومات حول الخدمات التي قد يتلقاها طفلي في المستقبل |
| لا | غير متاكد | نعم | **هل تحتاج الى مساعدة في كل مما يلي:** |
| 🞎 | 🞎 | 🞎 | زوجتي/ شرح حالة طفلي لوالدي أو والدي زوجي |
| 🞎 | 🞎 | 🞎 | شرح حالة طفلي لإخوته |
| 🞎 | 🞎 | 🞎 | معرفة كيفية الرد عندما يسأل الأصدقاء أو الجيران أو الغرباء عن طفلي |
| 🞎 | 🞎 | 🞎 | شرح حالة طفلي للأطفال الآخرين |
| 🞎 | 🞎 | 🞎 | العثور على مواد للقراءة عن العائلات الأخرى التي لديها طفل مثل طفلي |
| 🞎 | 🞎 | 🞎 | تحديد مكان جليسات الأطفال أو الرعاية المؤقتة المستعدات والقادرات على رعاية طفلي |
| 🞎 | 🞎 | 🞎 | تحديد موقع برنامج الرعاية النهارية أو مرحلة ما قبل المدرسة لطفلي |
| 🞎 | 🞎 | 🞎 | الحصول على الرعاية المناسبة لطفلي في المسجد |
| 🞎 | 🞎 | 🞎 | الاجتماع والتحدث مع الآباء الآخرين الذين لديهم طفل مثل طفلي |
| 🞎 | 🞎 | 🞎 | العثور على طبيب يمكنه فهم احتياجاتي واحتياجات طفلي |
| 🞎 | 🞎 | 🞎 | تحديد موقع طبيب اسنان الذي سوف يرى طفلي |
| 🞎 | 🞎 | 🞎 | لقاء مع مستشار (أخصائي نفسي أو أخصائي اجتماعي أو طبيب نفسي) |
| 🞎 | 🞎 | 🞎 | المزيد من الوقت للتحدث مع معلم طفلي أو المعالج |

**Research questionnaire (Scored version)**

**First:** Demographics

| **Age (years)** |  |
| --- | --- |
| **Relation to the child** | 🞎 Father 🞎 Mother |
| **Marital status** | 🞎 Married 🞎 Divorced/widowed |
| **Education** | 🞎 No formal education  🞎 Elementary school  🞎 Middle school  🞎 High school  🞎 Bachelor or above |
| **Residence** | 🞎 Urban 🞎 Rural |
| **No. of children** |  |
| **No. of autistic children** |  |
| **Consanguinity** | 🞎 Yes 🞎 No |
| **Child age (years)** |  |
| **Child gender** | 🞎 Male 🞎 Female |
| **Presence of comorbid conditions** | 🞎 Yes 🞎 No |
| **Type of comorbidity** |  |

**Second:** Family needs scale (FNS)

| **Do you need support in getting information on each of the following?** | **No** | **Not sure** | **Yes** |
| --- | --- | --- | --- |
| How children **grow and develop** | 1 | 2 | 3 |
| How to **play or talk** with my child | 1 | 2 | 3 |
| How to **teach** my child | 1 | 2 | 3 |
| How to handle my child’s **behaviour** | 1 | 2 | 3 |
| Information about **any condition or disability** my child might have | 1 | 2 | 3 |
| Information about **services** that are presently available for my child | 1 | 2 | 3 |
| Information about services my child might receive **in the future** | 1 | 2 | 3 |
| **Do you need support with each of the following** | **No** | **Not sure** | **Yes** |
| Explaining my child’s condition to **my parents or my spouse’s parents** | 1 | 2 | 3 |
| Explaining my child’s condition to **his or her siblings** | 1 | 2 | 3 |
| Knowing how to respond when **friends, neighbors, or strangers** ask questions about my child | 1 | 2 | 3 |
| Explaining my child’s condition to **other children** | 1 | 2 | 3 |
| Finding **reading material** about other families who have a child like mine | 1 | 2 | 3 |
| Locating **babysitters or respite care** who are willing and able to care for my child | 1 | 2 | 3 |
| Locating **a daycare program or preschool** for my child | 1 | 2 | 3 |
| Getting appropriate care for my child in **a mosque** | 1 | 2 | 3 |
| **Meeting & talking** with other parents who have a child like mine | 1 | 2 | 3 |
| **Locating a doctor** who can understand me and my child’s needs | 1 | 2 | 3 |
| **Locating a dentist** who will see my child | 1 | 2 | 3 |
| Meeting with a **counselor (psychologist, social worker or psychiatrist)** | 1 | 2 | 3 |
| More time to talk to my child’s **teacher or therapist** | 1 | 2 | 3 |
